# Microtubule encounter-based catastrophe in Arabidopsis cortical microtubule arrays

**DOI:** 10.1186/s12870-016-0703-x

**Published:** 2016-01-16

**Authors:** Zhihai Chi, Chris Ambrose

**Affiliations:** Department of Biology, University of Saskatchewan, 112 Science Place, Saskatoon, SK S7N 5E2 Canada

**Keywords:** Arabidopsis, Microtubule, Cytoskeleton, CLASP, Pavement epidermal cell, Microtubule-associated protein, *clasp-1* mutant

## Abstract

**Background:**

The cortical microtubules (CMTs) that line the plasma membrane of interphase plant cells are extensively studied owing to their importance in forming cell walls, and their usefulness as a model system for the study of MT dynamic instability and acentrosomal MT organization. CMTs influence the orientation and structure of cellulose microfibrils in the cell wall by cooperatively forming arrays of varied patterns from parallel to netted. These CMT patterns are controlled by the combined activities of MT dynamic instability and MT-MT interactions. However, it is an open question as to how CMT patterns may feedback to influence CMT dynamics and interactions.

**Results:**

To address this question, we investigated the effects of CMT array patterning on encounter-based CMT catastrophe, which occurs when one CMT grows into another and is unable to cross over. We hypothesized that the varied CMT angles present in disordered (mixed CMTs) arrays will create more opportunities for MT-MT interactions, and thus increase encounter-based catastrophe rates and distribution. Using live-cell imaging of Arabidopsis cotyledon and leaf epidermal cells, we found that roughly 87 % of catastrophes occur via the encounter-based mechanism, with the remainder occurring without encounter (free). When comparing ordered (parallel) and disordered (mixed orientation) CMT arrays, we found that disordered configurations show higher proportions of encounter-based catastrophe relative to free. Similarly, disordered CMT arrays have more catastrophes in general than ordered arrays. Encounter-based catastrophes were associated with frequent and sustained periods of pause prior to depolymerization, and CMTs with tight anchoring to the plasma membrane were more prone to undergo encounter-based catastrophe than weakly-attached ones. This suggests that encounter-based catastrophe has a mechanical basis, wherein MTs form physical barriers to one another. Lastly, we show that the commonly used measure of catastrophe frequencies (F_cat_) can also be influenced by CMT ordering and plasma membrane anchoring.

**Conclusions:**

Our observations add a new layer of complexity to our current understanding of MT organization in plants, showing that not only do individual CMT dynamics influence CMT array organization, but that CMT organization itself has a strong effect on the behavior of individual MTs.

## Background

Microtubules (MTs) are polymers of α/β-tubulin heterodimers that self-assemble into polar filaments with a fast-growing end, the plus end, and a relatively stable end, the minus end [1]. MTs play diverse roles at all stages of the eukaryotic cell cycle; guiding cell division, expansion and morphogenesis. To accomplish their varied tasks, MTs group together to form specialized arrays that are continuously remodeled by a process termed dynamic instability, wherein individual MT ends switch stochastically between growth and shrinkage through GTP hydrolysis [1, 2]. Transitions from growth to shrinkage are termed catastrophe, and the transitions from shrinkage to growth are called rescue [3]. These parameters are biochemically modulated by MT-associated proteins (MAPs), which perform a variety of functions including stabilizing MTs through promoting polymerization of tubulin [4, 5], facilitating MT-MT interactions (e.g. crosslinking) [6, 7], and destabilizing MTs through increasing depolymerisation rate or severing MTs [5, 8].

To orchestrate MT organization on a cellular scale, MT-organizing centers (MTOCs) are important structures that provide biochemical, mechanical, and positional information. By nucleating and tethering MTs, MTOCs create polarized groups of microtubules such as spindles and interphase cytoplasmic arrays. MTOCs are varied and ubiquitous throughout eukaryotes and include centrosomes in animal cells, spindle pole bodies in fungi [9], basal bodies in flagellated cells [9], the nuclear envelope in plants [10–17], and plastid in some algae [15]. However, in many cells, MTOCs are absent but MTs still obtain global ordering through self-organization, a process wherein global order emerges in a system from interactions between individual elements [18, 19]. This “acentrosomal” MT organization is intensely studied in the interphase cortical array of plants, which consists of MTs that are laterally attached to the plasma membrane and undergo continuous dynamic behaviours. These Cortical MTs (CMTs) arrange in various orientations ranging from random/netted, parallel, and highly bundled. By guiding cellulose synthase complexes, CMT organization influences cell wall microfibril patterning, which in turn guides cell expansion patterns [20]. Since CMTs are restricted to the plane of the plasma membrane and highly dynamic, the probability of interactions between individual CMTs is much higher than MTs that are free to roam in three dimensions. Indeed numerous types of MT-MT interactions have been documented and shown to influence CMT arrangement [19, 21, 22]. These MT-MT interactions include bundle formation, collision-induced catastrophe [21], severing [23, 24], and nucleation of MTs from pre-existing MTs [25–27].

With respect to bundle formation and collision-induced catastrophe, when a growing CMT encounters another CMT, the relative angle between the two MTs determines whether the incoming MT will form a bundle with the barrier CMT or undergo catastrophe [21]. Specifically, when the contact angle between a growing plus end and the barrier MT is a small angle (for example, less than 40°), the plus ends reorients and continues growth alongside the barrier MT, forming a bundle. But when the incoming MT contacts the barrier MT more directly (i.e. at a large angle), the MT either depolymerizes (encounter-based catastrophe) or crosses over. From this, Dixit and Cyr developed a model for self-organization of CMTs into parallel arrays, wherein bundling speeds up parallel CMT formation, and encounter-based catastrophe favors elimination of CMTs with orientations not parallel to the predominant CMT orientation [21]. Using quantitative experimental data from CMT dynamics, numerous computer simulations and mathematical models have supported this model, but the relative contributions of bundling and encounter-based catastrophe are less clear [21, 28–36].

Whether CMT encounter-based catastrophe results from biochemical and/or mechanical mechanisms (or both) is not known. Several catastrophe-inducing MAPs have been found in plants, including kinesin-13A, MAP18 and ARK1kinesin [37–39]. However, since CMT catastrophe also occurs without MT-MT encounter, it is unclear as to whether these MAPs have any specificity/preference for collision-induced catastrophe. In terms of mechanical forces, it is known that catastrophe can occur when a growing MT encounters a physical barrier. Specifically, the continued addition of tubulin subunits to the stalled MT plus end generates compressive force along the MT axis, which can induce MT bending and catastrophe [40–43]. In addition to MT-MT encounters, polymerization against cell edges can also induce CMT catastrophe [44, 45].

Given that MT-MT encounter outcomes are angle-dependent, it is possible that the highly variable arrangements of CMTs can influence rates of catastrophes, which may then feedback to CMT arrangements. In this study, we sought to investigate: (1) if CMT arrangement can influence catastrophe rates and spatial distribution; and (2) what the mechanistic underpinnings of encounter-based catastrophe are. To address these questions, we performed detailed quantifications of CMT arrangements, catastrophe types (encounter-based or free-based), spatial distributions, crossovers, and CMT-cortex attachment levels. We provide evidence for a strong contribution of force-induced catastrophe, and show that indeed CMT arrangement can influence catastrophe types and frequencies.

## Results

### Encounter-based catastrophes constitute the dominant source of CMT catastrophe

**Fig. 1:**
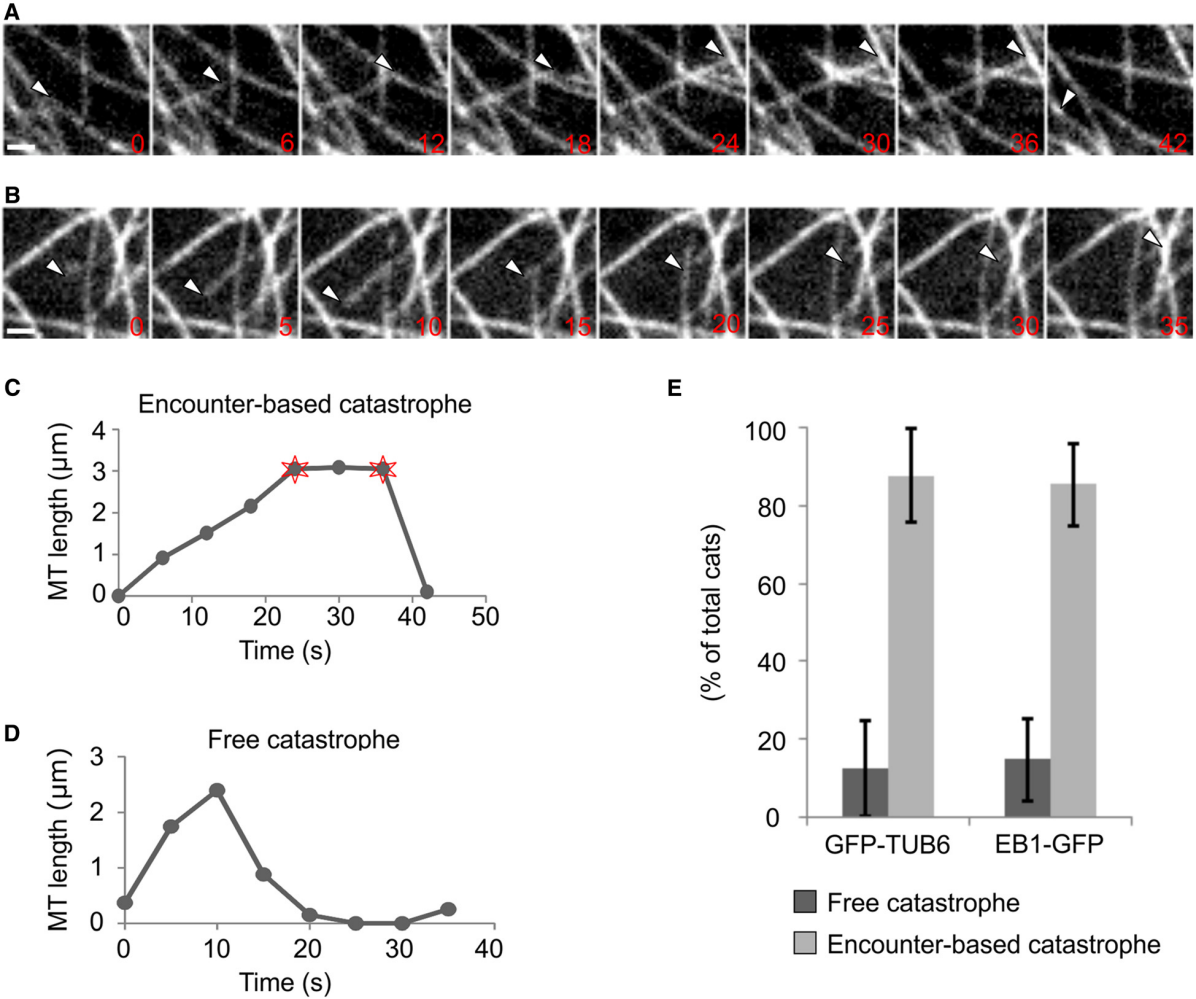
Two types of catastrophe and their frequencies of occurrence. **a** Encounter-based catastrophe and **b** free catastrophe. *White arrowheads* mark CMT plus ends. Numbers indicate time in seconds. **c**, **d** MT length-changes over time, corresponding to the tracked MTs in (**a** and **b**), respectively. The stars mark the start and end of a pause. **e** Encounter-based catastrophe is the dominant type of CMT catastrophe. Data were obtained using GFP-TUB6 and EB1-GFP as MT reporters. For GFP-TUB6, *n* = 48 cells, 376 catastrophe events; for GFP-EB1b, *n* = 20 cells, 245 catastrophe events. Bars: **a**, **b** = 1 μm

To visualize CMT dynamics, we performed confocal imaging using cotyledons and leaves of Arabidopsis seedlings constitutively expressing GFP fused to Arabidopsis TUBULIN BETA6 (GFP-TUB6) [46]. All data shown represent the combined data from leaves and cotyledons. The sharp cell edges present in meristematic and unexpanded cells are known to induce catastrophe when MTs grow into them [44], so in the current study we used mature epidermal cells in order to remove any potential cell edge effects on catastrophe behavior. The large size and rounded cell edges of these cells allowed sampling of large and relatively flat central areas of the outer cell face that were typically tens of microns away from nearby edges. For characterization of catastrophe types, we classified MT catastrophe into encounter-based catastrophe (Fig. 1a, c) or free catastrophe (Fig. 1b, d). Notably, we found that 87.6 % of catastrophes in leaves and cotyledons were encounter-based, with the remaining 12.4 % being not associated with encounter (Fig. 1e). The quantification of another line expressing *35S:GFP-EB1b* showed a similar trend, with 85.3 % of catastrophes being encounter-based (Fig. 1e).

### CMT organization influences the type and spatial distribution of catastrophes

**Fig. 2:**
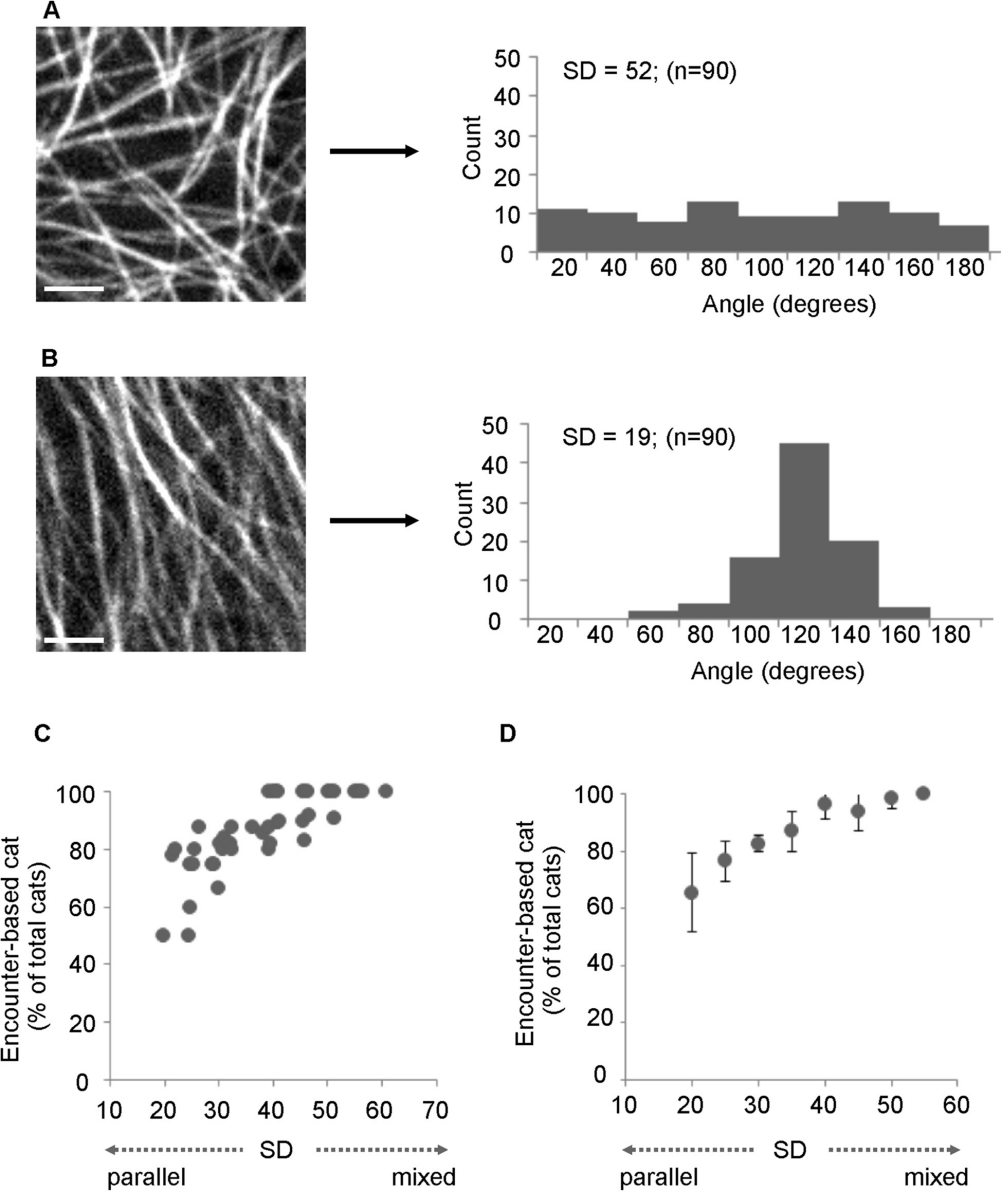
The ratio of encounter-based to free catastrophe varies with CMT arrangement. Examples of disordered (**a**) and highly ordered CMTs (**b**). Histograms show CMT angle distributions corresponding to the images. **c**, **d** Disordered CMT arrays have a higher proportion of encounter-based catastrophes relative to free catastrophes. CMT order is expressed as the SD of CMT angles. **c** Un-binned data from individual cells (i.e. each point represents one cell); **d** data from (**c**) binned in five SD units. Bars: **a**, **b** = 2.5 μm

Based on these data, we hypothesized that compared to ordered (parallel) arrays, a highly disordered CMT array will provide more opportunities for CMT collision, and will thus have a larger proportion of encounter-based catastrophes compared to free catastrophes (i.e. a higher ratio of encounter-based to free). To test this, we measured CMT angles within parallel and disordered arrays (see Methods section for CMT angle measurements) and assessed the relative frequency of encounter-based catastrophes (i.e. % encounter-based to total catastrophes). As a measure of array order, we used the standard deviation of CMTs angles. With this method, parallel arrays show smaller standard deviations in CMT angle than net-like arrays. This is illustrated in Fig. 2, which shows an example of a disordered array (SD = 52) (Fig. 2a) and an example of an ordered array (SD = 19) (Fig. 2b). To ensure specificity of our data, SD and catastrophe ratios were measured on a cell-by-cell basis. We sampled 48 cells with varying degrees of order, and found a correlation between CMT array disorder and encounter-based catastrophes (Fig. 2c and d). Specifically, the more disordered a CMT array is, the higher the proportion of encounter-based catastrophes is. We observed a similar trend using *35S:GFP-EB1b* lines (data not shown). These results indicate that CMT arrangement is an important determinant of catastrophe type.

**Fig. 3:**
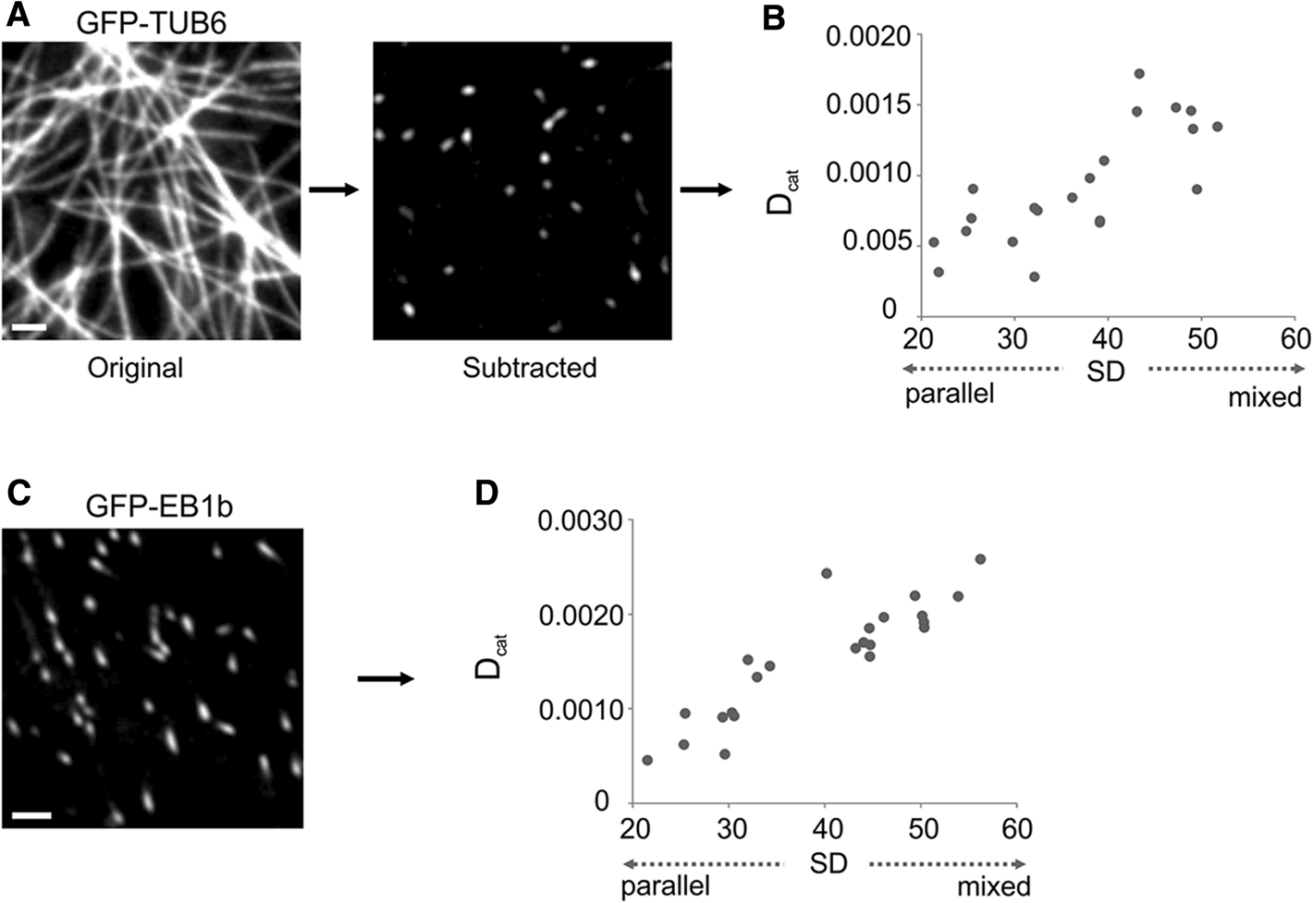
Density of catastrophe (D_cat_) varies with CMT arrangement. **a** Example of subtracted image used for measurements to count growing plus ends with GFP-TUB6. **b** Density of catastrophe (D_cat_) increases with increasing CMT array disorder (expressed as SD of CMT angles). Measurements derived from GFP-TUB6 subtraction method. **c** Example of GFP-EB1b image used to count growing plus ends. **d** GFP-EB1b data shows a similar trend to GFP-TUB6 in (**b**). Bars: **a**, **c** = 2 μm

Given this organizational influence on catastrophe type, we hypothesized that disordered CMT arrays will also have a higher number of catastrophe events in general (i.e. per unit area and time). We term this property as Density of Catastrophe (D_cat_) in order to clearly distinguish it from the dynamic instability parameter Catastrophe Frequency (F_cat_). Proper quantification of D_cat_ presents a number of challenges. For example, simply measuring catastrophes per cellular area is problematic because the MT density itself within a given area would be a major determinant of density of catastrophe. Similarly, even normalizing for the area occupied by MTs is biased due to the fact that many MTs are very long, bundled, and stable, such that they span the entire region of observation and do not show observable MT end dynamics during the duration of the observation period. Based on these caveats, we chose to normalize density of catastrophe by expressing it as a function of the number of growing plus ends present within the observational region. To obtain simple and accurate counts of growing plus ends, we employed a subtractive technique similar to that used by Burnette et al. 2011 [47]. By subtracting pixel values between sequential time frames, only areas occupied by new MT growth are shown in the output image (Fig. 3a). Using this method, we indeed found a positive correlation between CMT disordering and density of catastrophe (Fig. 3b). We found a similar trend using GFP-EB1b lines as an indicator of plus end density (MT sidewall labeling was enough to detect the presence or absence of a barrier MT for each catastrophe) (Fig. 3c, d). These data indicate that CMT organization can influence the type and density of catastrophes.

### Strong MT-cortex attachment favors encounter-based catastrophe

**Fig. 4:**
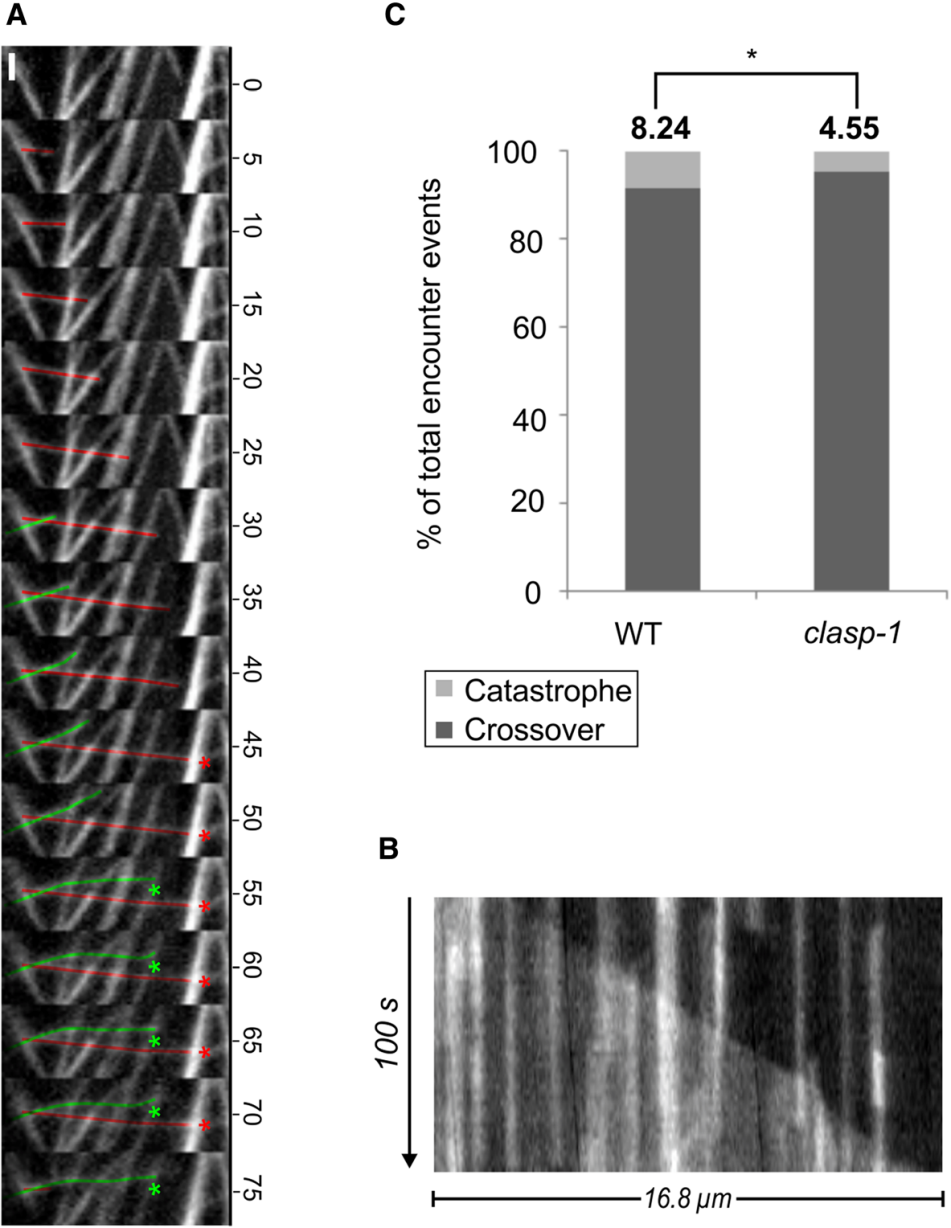
Encounter-based catastrophe is related to CMT-cortex attachment. **a** Example of cross-over and encounter-based catastrophe in WT. *Red line* marks several cross-over events before pause and encounter-based catastrophe. *Green line* marks a detachment event as described in Results. Numbers indicate time in seconds. Both asterisks indicate pause events. Bars: **a** = 2 μm. **b** Kymographs showing CMT with several cross-over events. **c** Quantification of crossover and encounter-based catastrophe. Asterisk indicates statistically significant difference of catastrophe frequency between WT and *clasp-1* when *P* < 0.05. *n* = 10 cells, 3675 encounter events

We next sought to investigate the mechanism governing encounter-based catastrophe. Given that MT polymerization against a physical barrier is known to induce MT bending and depolymerization [44, 48], and that the degree of CMT-cortex attachment influences the outcome of MT-MT interactions by imposing constraints on lateral motions of the MT polymer [30, 49], we hypothesized that when a weakly-attached CMT encounters a barrier MT, it will be more prone to lateral motions such as bending and detachment, which act to relieve the axial compression on the incoming MT, and hence reduce the probability of depolymerization. To test this, we quantified CMT behaviour in *clasp-1* mutants, which show decreased CMT-cortex attachment [49]. To quantify the frequencies of encounter-based catastrophe, we classified encounter events as either cross-over or catastrophe (MT-MT bundling events were excluded). In general, cross-overs were far more numerous than catastrophes in both genotypes, in agreement with previous findings in Arabidopsis [23]. Figure 4a shows two examples of MT crossovers, one of which grows in a straight line and eventually undergoes pause and encounter-based catastrophe (red), and another which shows partial detachment and swinging of the growing end as it grows across other MTs (green). This MT appears to be less anchored to the cortex because after it re-attaches, it encounters another MT and stalls, during which time it bends (presumable due to force of continued polymerization at the stalled plus end). Figure 4b shows a kymograph of an individual MT growing across several other MTs (which appear as vertical lines in the kymograph).

For quantification of crossover and catastrophes, measurements were normalized using the ratio of encounter-based catastrophe to total encounter events (i.e. catastrophe + cross-over). We found that CMTs in *clasp-1* are more prone to cross over barrier MTs than wild type, and are less likely to undergo catastrophe (Fig. 4c). These data support the idea that strongly-attached MTs are prone to catastrophe because they are restricted to any lateral movement (which would dissipate axial compression), and are unable to detach and cross over the barrier MT.

### Encounter-based catastrophes exhibit a pause in growth before catastrophe

**Fig. 5:**
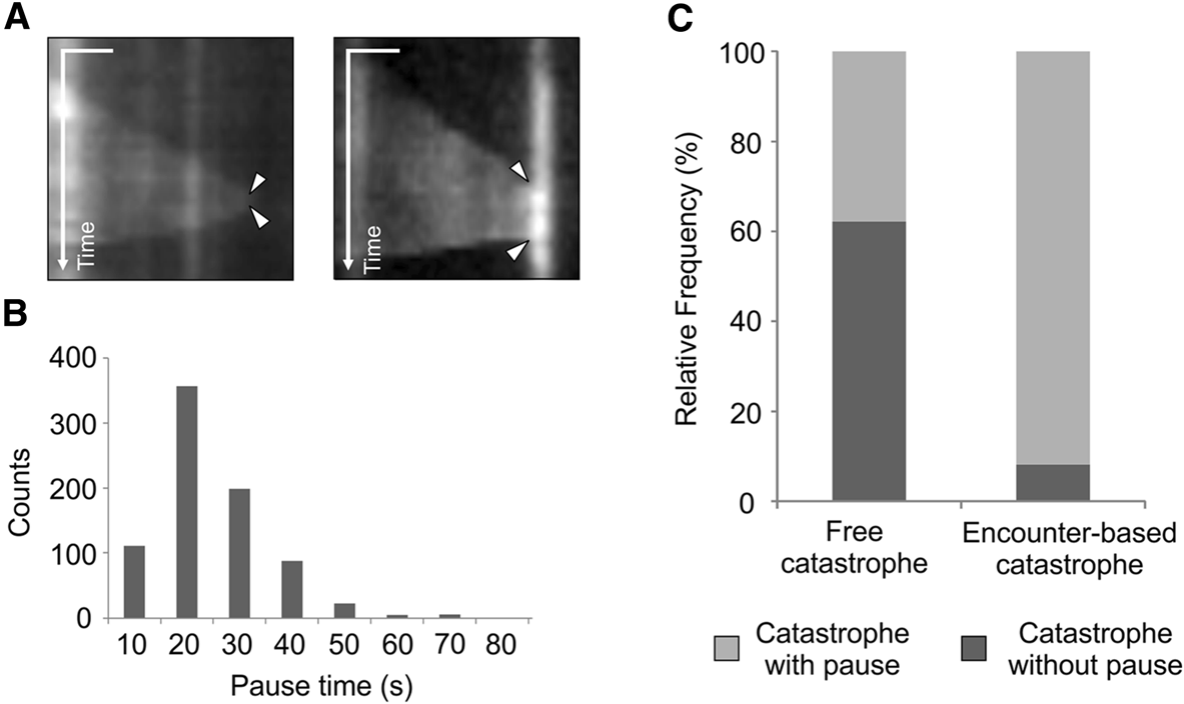
CMTs with encounter-based catastrophe pause upon encounter with the barrier MT. **a** Kymographs showing free catastrophe without pause (*left panel*) and encounter-based catastrophe with pause (*right panel*). The *arrowheads* mark the start and the end of contact. Bars: The kymographs display a period of 76 s, scale bar = 1 μm. **b** Histogram showing the distribution of pausing times for MTs that undergo encounter-based catastrophe. The pausing time ranges from 10 to 80 s, and average time is 19.23 s. **c** Encounter-based catastrophes are preceded by pause more frequently than are free catastrophes. *n* = 107 cells, 119 free catastrophe and 1196 encounter-based catastrophe events

During our analysis of catastrophe events, we noticed that the growth of an incoming CMT often pauses upon encounter with a barrier MT and is followed by catastrophe and depolymerization. Figure 5a shows kymographs of free catastrophes without pause (left panel) and encounter-based catastrophe with pause (right panel). As shown in the histogram in Fig. 5b, the time spent in pause state for encounter-based catastrophe event varies considerably, with a mean of 19 ± 11 s, and a maximum of 80 s. For subsequent analysis, we defined pause as a state during which no observable MT growth is detectable for more than 5 s. Quantification of pause events revealed that 91.7 % of encounter-based catastrophe events were preceded by pause (Fig. 5c). In contrast, only 37.8 % of free catastrophes were preceded by pause, and the mean time for pause is 12 ± 6 s (~34 % less than for encounter-based pauses) (Fig. 5c). Taken together, these data show that encounter of a growing CMT plus-end with a barrier MT greatly enhances the likelihood and duration of pause prior to depolymerization.

### CMT instability dynamics for catastrophe frequency vary with array organization

Having found that CMT-cortex attachment and CMT organization both have effects on CMT catastrophes, we hypothesized that these factors may lead to inconsistencies in standard measurements of catastrophe frequencies (F_cat_). To test this, we measured and compared F_cat_ values between disordered and parallel arrays; as well as between weakly-attached and strongly-attached CMT arrays (i.e. *clasp-1* vs WT) (Results summarized in Table 1). F_cat_ was calculated using the total number of catastrophe events divided by the total time spent in growth and pause, and only CMTs that show catastrophe at any point during its observation were used for measurements [21, 50, 51]. Notably, we found that CMTs showing free-type catastrophes have a significantly higher F_cat_ (0.030 ± 0.009 events/s) compared to those having encounter-based catastrophes (0.021 ± 0.002 events/s) (Table 1). However, this difference is heavily biased from the longer durations of pause associated with encounter-based catastrophes (i.e. longer pauses increase total measured MT growth times, and thus decreased F_cat_). Despite these differences, when comparing F_cat_ between disordered CMT arrays (defined as arrays with SDs between 50 and 60) and well-ordered (defined as arrays with SDs between 20 and 30), we found no significant difference in F_cat_ values (disordered = 0.022 ± 0.002 events/s; Ordered = 0.021 ± 0.002 events/s) (Table 1). This suggests that although free catastrophes do have higher average F_cat_ values than encounter-based catastrophe, they are not numerous enough to significantly influence overall cellular F_cat_ measurements. However, when assessing the influence of CMT-cortex attachment on F_cat_, we found that wild-type cells have a significantly higher F_cat_ (0.021 ± 0.001 events/s) than *clasp-1* (0.014 ± 0.001 events/s) (Table 1). This is consistent with the above observations of WT CMTs being less likely to cross over the barrier MT (Fig. 4c).

**Table 1:** Catastrophe frequency varies between different catastrophe types and cell types. Values are means ± s.d. For catastrophe frequencies, *n* = 12 cells and 191 catastrophe events; for F_cat_ in both WT and *clasp*, *n* = 10 cells and 234 catastrophe events

CMT catastrophe frequencies (events/s)	
General catastrophe F_cat_	0.022 ± 0.002
Free catastrophe F_cat_	0.030 ± 0.009
Encounter-based catastrophe F_cat_	0.021 ± 0.002
Less organized CMT catastrophe F_cat_	0.021 ± 0.003
Well organized CMT catastrophe F_cat_	0.022 ± 0.002
General catastrophe in WT F_cat_	0.021 ± 0.001
General catastrophe in *clasp* F_cat_	0.014 ± 0.001

## Discussion

Since the first characterization of plant CMT dynamic instability [2], several studies have made dynamic measurements using a variety of cell types, mutants, and drug treatments to investigate the mechanisms of CMT organization [4, 5, 20, 46, 50–59]. From these studies, several types of MT-MT interactions were discovered and have emerged to be major players in defining CMT organization and behaviour. Our findings build on this, showing that CMT organization can itself feedback to influence CMT interactions. The observation that ~87 % of CMT catastrophes are associated with MT-MT encounter indicate that MT catastrophe in particular is heavily influenced by MT-MT interactions and CMT array organization. Observations by Zhang et al. (2013) and Wightman et al. (2007) that MTs frequently depolymerize following their katanin-dependent severing at MT-MT crossover points suggests that severing rates may also be influenced by overall CMT organization (since disorganized arrays have more MT-MT crossover points) [23, 60].

Based on our findings that MT organization can influence the catastrophe properties of individual MT is important to consider when assessing the in vivo functions of plant MAPs because it may confound assumed measurements of biochemical activities. This is illustrated in the current study by our *clasp-1* mutant data. Without taking into account MT-MT influences on dynamic instability, our observations of reduced F_cat_ in *clasp-1* mutants could be interpreted to indicate that CLASP functions biochemically to *induce* MT catastrophe, thereby acting as a MT de-stabilizer. However, numerous studies have shown that CLASP stabilizes MTs via promotion of pause and rescue in animals and fungi [45, 61, 62]. This apparent contradiction can be reconciled by our data, which suggest that the *clasp-1* reduction in F_cat_ results at least in part from the enhanced MT cross-overs associated with weakly cortex-bound CMTs. CLASPs generally stabilize MTs specifically in localized regions of the cell such as cell edges [29, 44], chromosomal kinetochores [63], and between overlapping regions of interpolar spindle microtubules in fission yeast [64]. Therefore, measurements from different parts of a given cell can also yield seemingly contradictory data.

With respect to the mechanism of encounter-based catastrophe, our data suggest that in plants, mechanical forces on MTs play a major role in catastrophe induction. This applies not only to CMT encounter-based catastrophe, but also to cell edge-induced catastrophe [44]. Thus, when considering the cell as a whole, the bulk of CMT catastrophes occur either at cell edges [44] or via encounter-based catastrophe (current study). In both cases, catastrophe is preceded by behaviours characteristic of MT polymerization against a physical barrier such as prolonged pause and bending [23, 43, 44, 65, 66].

## Conclusions

Studies on CMT dynamics and organization in plants generally have focused on the mechanisms by which CMT behavior influences cellular CMT organization. The data presented in the current study show that the organization of CMTs within a cell also has strong influences on CMT dynamic behavior. Thus, a dynamic interplay exists between cellular CMT organization and individual CMT dynamics, indicating feedback between the two elements.

## Methods

### Plant materials and growth conditions

Transgenic Arabidopsis thaliana (Columbia-0) and *clasp-1* lines expressing *35S:TUB6-GFP* [49, 67], as well as WT plants expressing *35S:GFP-EB1b* [68] were used for measurements. Prior to plating, all seeds were stored in the dark at 4 °C for 2 d. Seeds were sterilized in 70 % ethanol, rinsed twice with sterile water, and plated onto Petri dishes containing ½ MS media, 1.0 % agar, and 1 % sucrose. Plates were wrapped with nescofilm (Azwell Inc.) and transferred to a 21 °C growth cabinet (with continuous light) and placed vertically. Young pavement cells of leaf and cotyledon cells were imaged at 3–4 days.

### Microscopy and image analysis

All observations were performed on living cells. Cotyledons/leaves were cut at their base, mounted on to slides in Perfluoroperhydrophenanthrene (PP11) [69] under coverslip and sealed with wax. Images were obtained via point-scan confocal microscopy (Zeiss Meta 510 with Zeiss Axiovert 200M microscope, 63X water immersion), TIRF (Olympus IX83 with ANDOR iXon Ultra EMCCD camera, 100X oil immersion), and structured light illumination (Zeiss Apotome mounted on Zeiss Axio Imager Z1 with AxioCam MRm, 63x oil immersion). Time-lapse intervals ranged from 2–5 s. Images were processed with Image J software (http://rsb.info.nih.gov/ij/), and figures were assembled using Corel Draw software.

### CMT angle and density measurements

For quantification of CMT array order, images were sampled by using five evenly-spaced horizontal rectangular boxes that were 2 μm high and as wide as the image. The narrow height was used to remove any ambiguity of MT angle that may arise due to MTs that are bent to varying degrees. Only regions of cells that were covered by a sampling box were measured, which helped remove selection bias when using large cells. MT angles were defined relative to the horizontal axis of the image. Using a second method to measure CMT angles produced similar results. For this, a central area of the cell (away from edges) was sampled for up to 30 MTs.

For quantification of SF_cat_, subtractive analysis of sequential time frames was performed by duplicating the time-lapse image twice, and removing one to two frames from the start of the first duplicate, and removing the same number of frames from the end of the second duplicate. Using the image calculator function of ImageJ, the duplicates were then subtracted, and the resulting image was used to count growing plus ends. Since catastrophes were measured for the entire time series, we sampled plus end numbers at four evenly spaced time-points throughout the series and averaged the counts. 21 cells were sampled in total.

## Abbreviations

CLASP: clip-associated protein
CMT: cortical microtubule
D_cat_: Density of Catastrophe
EB1b: end-binding protein 1b
F_cat_: frequency of catastrophe
GFP: green fluorescent protein
MAP: microtubule-associated protein
MT: microtubule
